# Keratinocytes sense and eliminate CRISPR DNA through STING/IFN-κ activation and APOBEC3G induction

**DOI:** 10.1172/JCI159393

**Published:** 2023-05-01

**Authors:** Mrinal K. Sarkar, Ranjitha Uppala, Chang Zeng, Allison C. Billi, Lam C. Tsoi, Austin Kidder, Xianying Xing, Bethany E. Perez White, Shuai Shao, Olesya Plazyo, Sirisha Sirobhushanam, Enze Xing, Yanyun Jiang, Katherine A. Gallagher, John J. Voorhees, J. Michelle Kahlenberg, Johann E. Gudjonsson

**Affiliations:** 1 Department of Dermatology, and; 2 Graduate Program in Immunology, University of Michigan, Ann Arbor, Michigan, USA.; 3 Department of Dermatology, Northwestern University, Chicago, Illinois, USA.; 4 Department of Dermatology, Xijing Hospital, Fourth Military Medical University, Xi’an, Shannxi, China.; 5 Department of Internal Medicine, Division of Rheumatology, University of Michigan, Ann Arbor, Michigan, USA.; 6 Department of Dermatology, Peking Union Medical College Hospital, Chinese Academy of Medical Sciences and Peking Union Medical College, Beijing, China.; 7 Section of Vascular Surgery, Department of Surgery,; 8 Department of Microbiology and Immunology, and; 9 Taubman Medical Research Institute, University of Michigan, Ann Arbor, Michigan, USA.

**Keywords:** Cell Biology, Dermatology, Cytokines, Gene therapy, Skin

## Abstract

CRISPR/Cas9 has been proposed as a treatment for genetically inherited skin disorders. Here we report that CRISPR transfection activates STING-dependent antiviral responses in keratinocytes, resulting in heightened endogenous interferon (IFN) responses through induction of IFN-κ, leading to decreased plasmid stability secondary to induction of the cytidine deaminase gene *APOBEC3G*. Notably, CRISPR-generated KO keratinocytes had permanent suppression of IFN-κ and IFN-stimulated gene (ISG) expression, secondary to hypermethylation of the *IFNK* promoter region by the DNA methyltransferase DNMT3B. JAK inhibition via baricitinib prior to CRISPR transfection increased transfection efficiency, prevented *IFNK* promoter hypermethylation, and restored normal IFN-κ activity and ISG responses. This work shows that CRISPR-mediated gene correction alters antiviral responses in keratinocytes, has implications for future gene therapies for inherited skin diseases using CRISPR technology, and suggests pharmacologic JAK inhibition as a tool for facilitating and attenuating inadvertent selection effects in CRISPR/Cas9 therapeutic approaches.

## Graphical abstract

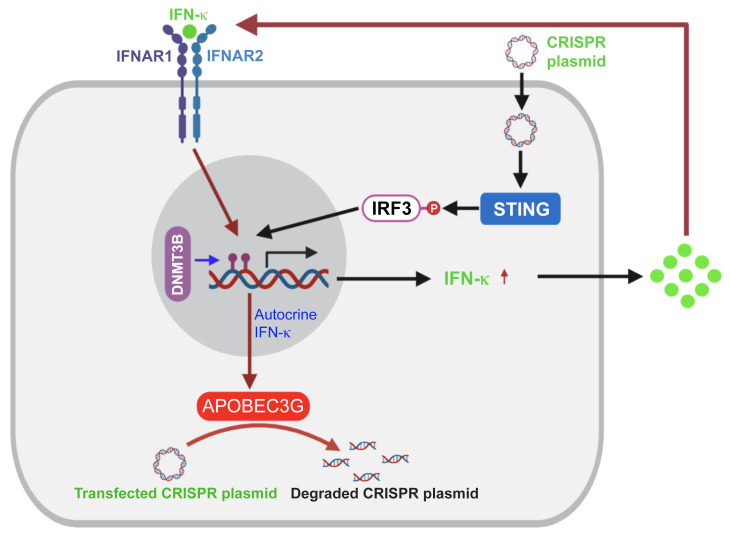

## Introduction

Keratinocytes (KCs) have long been recognized as one of the most difficult cell types to transfect (1, 2), but the mechanisms behind this resistance have remained unknown. KCs are the major cellular constituent of the epidermis, which acts as the primary interface between the body and external agents such as bacteria and viruses. Beyond their critical contribution to the epidermis as a physical barrier, KCs also have a very active immunological role through a range of pattern receptors and the ability to secrete various cytokines (3).

The prokaryote-derived clustered regularly interspaced short palindromic repeats (CRISPR)/Cas9 technology has transformed our ability to manipulate specific DNA and RNA sequences in living cells (4). The CRISPR/Cas9 system relies on guide RNAs (gRNAs) for targeting specificity and functions by generating targeted DNA breaks that stimulate repair by various endogenous mechanisms. CRISPR technology can be used for the insertion or deletion of small DNA segments through the nonhomologous end-joining pathway or single-base editing through homology-directed repair (4). CRISPR/Cas9 has increasingly been applied to gene therapy for a wide range of human diseases, including retinitis pigmentosa (5), Duchenne muscular dystrophy (6), and monogenic dominant diseases such as epidermolysis bullosa (7–10), a devastating, frequently lethal disease caused by mutations in structural genes involved in epidermal function. The role of KCs’ intrinsic resistance to CRISPR/Cas9 transfection has not been addressed, nor has the role of KCs’ intrinsic antiviral machinery, in mediating transfection resistance in KCs. Addressing this in KCs would greatly facilitate future efforts toward efficient gene correction in inherited epidermal diseases.

## Results

### KCs have constitutive STING-dependent IFN activity and are highly resistant to CRISPR/Cas9 transfection.

**Figure 1:**
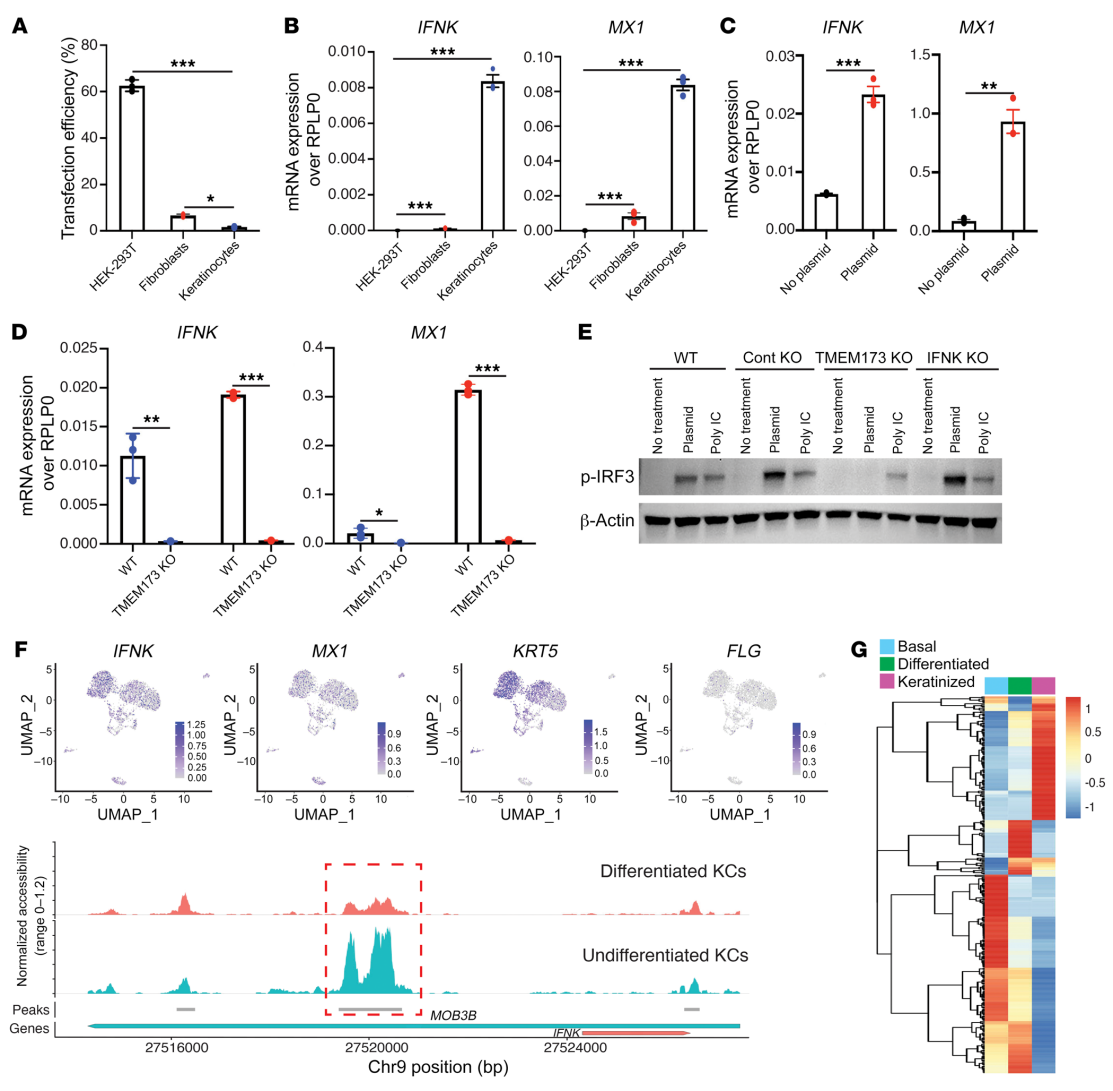
Keratinocytes activate type I IFN responses and sense foreign DNA through the STING pathway and are resistant to CRISPR/Cas9 transfection. (**A**) Comparison of transfection efficiency in keratinocytes (KCs), fibroblasts (FBs), and HEK293T cells (*n* = 3). (**B**) *IFNK* and *MX1* mRNA expression in KCs, FBs, and HEK293T cells (*n* = 3). *RPLPO*, large ribosomal protein mRNA (loading control). (**C**) Induction of *IFNK* and IFN-stimulated gene (ISG) *MX1* by CRISPR plasmid transfection (*n* = 3). (**D**) *IFNK* and *MX1* expression in WT and *TMEM173*-KO (STING-KO) KCs treated with CRISPR plasmid (*n* = 3). Bars with blue dots indicate no treatment; bars with red dots indicate CRISPR plasmid treatment. (**E**) p-IRF3 Western blot of plasmid-treated KO KCs. (**F**) scATAC-seq from healthy human epidermis shows the overlap between *IFNK*, *MX1*, and *KRT5* open chromatin regions (upper panels). Chromatin accessibility in the *IFNK* promoter region is greater in undifferentiated KCs compared with differentiated KCs (indicated by the dotted red box, lower panel). (**G**) Heatmap of type I ISGs from scRNA-seq data of healthy human epidermis shows localization of majority of ISGs in the basal epidermal compartment (*n* = 3). In the heatmap, red indicates higher expression and blue denotes the lower expression of type I ISGs. Data in **A**–**E** are represented as mean ± SEM. *P < 0.05; **P < 0.01; ***P < 0.001 by 1-way ANOVA with Tukey’s test (**A** and **B**) or 2-tailed Student’s *t* test (**C** and **D**).

To determine the transfection resistance of KCs, we compared transfection efficiency in human embryonic kidney 239T (HEK293T) cells, dermal fibroblasts, and KCs. Transfection efficiency using a liposome-based system was greater than 60% in HEK293T cells, 7% in fibroblasts, and only 1% in KCs (Figure 1A and Supplemental Figure 1A; supplemental material available online with this article; https://doi.org/10.1172/JCI159393DS1). To further understand these differences in transfection efficiency, we observed that KCs have constitutive expression of the IFN-stimulated gene (ISG) *MX1*, whereas this was seen in neither fibroblasts nor HEK293T cells. This corresponded to increased expression of the type I IFN, IFN-κ, which was detected only in KCs, and not fibroblasts or HEK293T cells (Figure 1B, Supplemental Figure 1B, and Supplemental Figure 2A). Notably, we observed a significant increase in both *IFNK* and *MX1* mRNA expression in KCs after CRISPR plasmid transfection (Figure 1C and Supplemental Figure 2B), suggesting that the CRISPR plasmid is recognized by intracellular nucleic acid sensors in KCs. *IFNB1* also showed induction in KCs following CRISPR transfection, although to a much lesser extent (Supplemental Figure 3).

The stimulator of IFN genes (STING) is known to control the induction of innate immune genes in response to the recognition of double-stranded DNA (dsDNA) (11). To address the role of STING in response to CRISPR transfection in KCs, we generated *TMEM173* (STING protein) KO in KCs (Supplemental Figure 4). In contrast to WT KCs, *TMEM173*-KO KCs completely abrogated both *IFNK* and *MX1* mRNA expression and the IFN response to CRISPR transfection (Figure 1D and Supplemental Figure 5A). STING activation results in the recruitment of the transcription factor IFN regulatory factor 3 (IRF3) and promotes phosphorylation of IRF3 (12) to activate type I IFNs and ISGs. We assessed phosphorylation of IRF3 (p-IRF3) by Western blotting in WT and KO KCs, including *TMEM173* and *IFNK* KOs. Whereas robust p-IRF3 was seen in WT, control KO, and *IFNK*-KO KCs, p-IRF3 was markedly reduced in the *TMEM173*-KO KCs upon CRISPR/Cas9 transfection (Figure 1E and Supplemental Figure 6). These data suggest that CRISPR/Cas9 transfection induces IFN-κ and ISGs in KCs through activation of the STING pathway. Notably, this activation of the STING pathway was not dependent on the constitutive activity of IFN-κ, as IRF-3 was robustly phosphorylated in *IFNK*-KO KCs. We next examined whether the upregulation of IFN-κ varied by KC differentiation state. IFN-κ has an established role in host defense against viral pathogens such as human papilloma viruses (HPVs) (13, 14), and nononcogenic HPV infections typically do not involve the basal layer of the epidermis (15) and are instead localized in the upper spinous layers (16). Consistent with a stronger IFN-κ response in the basilar KCs, both *TMEM173*/STING and *IFNK* mRNA expression was highest in undifferentiated KRT5^+^ basal epithelium, in contrast to more differentiated KCs (FLG^+^) and corresponded to open chromatin areas around the *IFNK* promoter, as shown by single-cell ATAC-seq (scATAC-seq) (Figure 1F and Supplemental Figure 7). Consistent with this observation, scRNA-seq of epidermal cells demonstrated that both IFN-κ and a majority of ISGs were primarily expressed in the basal layer of the epidermis (Figure 1G). These observations would predict that KCs in the basal layer of the epidermis would be more resistant to CRISPR/Cas9 transfection.

### STING-dependent induction of the cytidine deaminase APOBEC3G restricts CRISPR/Cas9 transfection efficiency in KCs.

**Figure 2:**
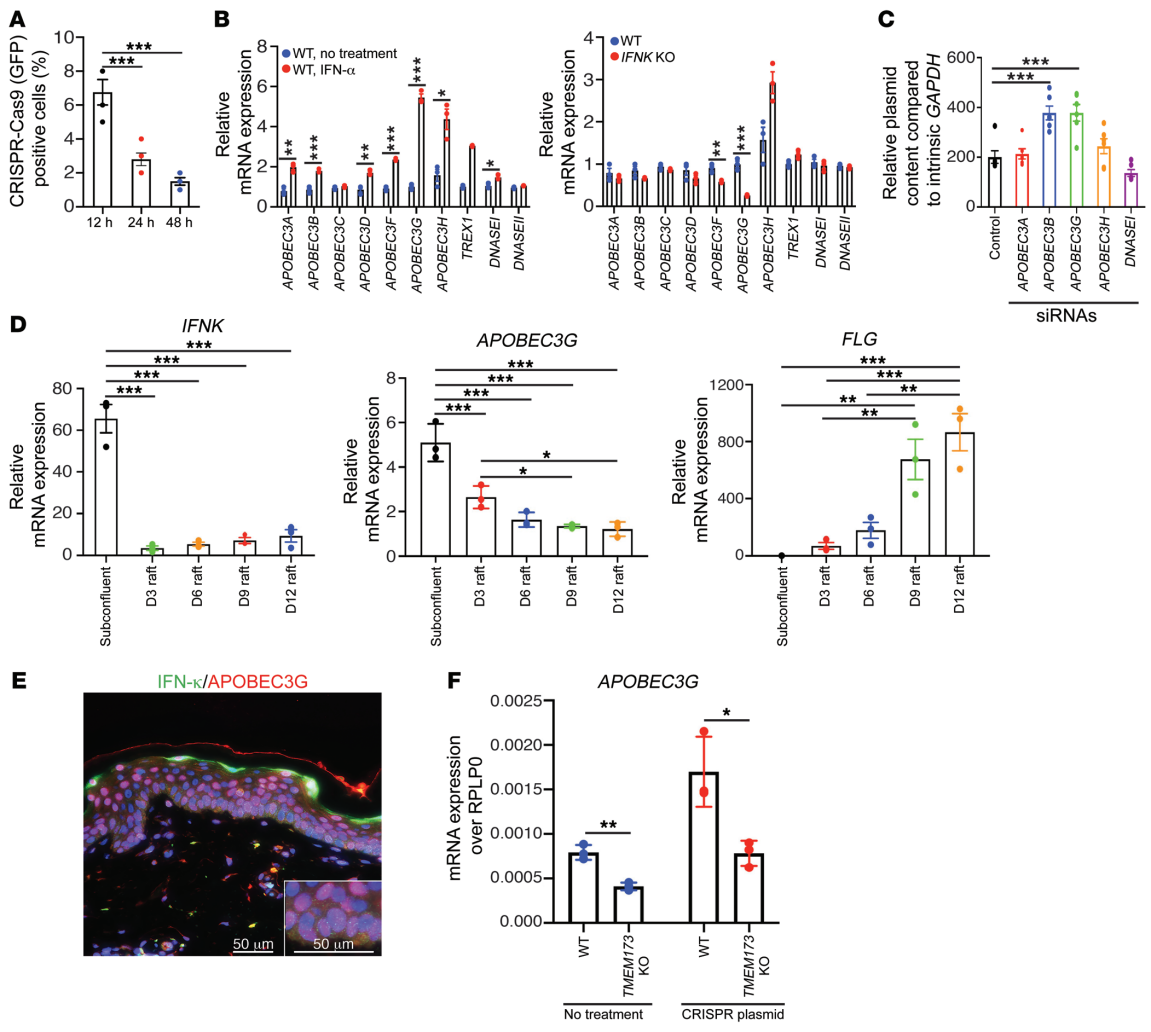
STING-dependent induction of the cytidine deaminase APOBEC3G restricts CRISPR/Cas9 transfection efficiency in keratinocytes. (**A**) Percentage of GFP-positive cells at different time points after CRISPR transfection (*n* = 3). (**B**) *APOBEC3*s’ mRNA expression in IFN-α–treated keratinocytes (KCs) and *IFNK*-KO KCs (*n* = 3). (**C**) CRISPR plasmid stability in KCs treated with *APOBEC3* siRNAs (*n* = 5). (**D**) Expression of *IFNK*, *APOBEC3G*, and *FLG* mRNA in subconfluent monolayer cultures and 3D epithelial raft cultures at different stages of differentiation (day 3 [D3] through D12) (*n* = 3). (**E**) APOBEC3G (red) and IFN-κ (green) immunostaining in healthy skin (*n* = 3). Scale bars: 50 μm. (**F**) *APOBEC3G* mRNA expression in *TMEM173*-KO KCs (*n* = 3). Data in **A**–**D** and **F** are represented as mean ± SEM. *P < 0.05; **P < 0.01; ***P < 0.001 by 1-way ANOVA with Tukey’s test (**A**, **C**, and **D**) or 2-tailed Student’s *t* test (**B** and **F**).

To determine whether the uptake of the CRISPR/Cas9 plasmid is defective in KCs, we measured the uptake and stability of CRISPR/Cas9 GFP-tagged plasmids in KCs at different time points after transfection. While CRISPR/Cas9 GFP^+^ was observed in approximately 6%–8% of KCs at early time points, this rapidly decreased to 1%–2% over a period of 48 hours (Figure 2A and Supplemental Figure 8A). This uptake followed by rapid disappearance suggests that KCs actively degrade the CRISPR plasmid shortly after transfection, and prior to the interaction of CRISPR/Cas9 with its DNA target. DNases such as DNase I and DNase II, along with the APOBEC3 protein family of cytidine deaminases, have been shown to mediate the clearance of foreign DNA from human cells (17–19). To assess the involvement of DNase I, DNase II, and APOBEC3 family members in the clearance of CRISPR/Cas9 plasmids from KCs after transfection, we used RNA-seq to compare the expression profiles for type I IFN–treated versus *IFNK*-KO KCs. While the majority of the APOBEC3 family members showed increased mRNA expression, only minor shifts were seen for *DNASE1*, and no changes were observed for *DNASE2* mRNA expression. Correspondingly, *IFNK*-KO KCs had decreased mRNA expression of 3 of the APOBEC3 members, *APOBEC3A*, *APOBEC3F*, and *APOBEC3G*, whereas only *APOBEC3H* was increased (Figure 2B). To determine the potential role of these 4 APOBEC3 members and DNase1 in CRISPR/Cas9 plasmid stability, we used siRNA to knock down each of the 4 *APOBEC3* genes and *DNASE1* in KCs. Only si*APOBEC3B* and si*APOBEC3G* treatments increased plasmid stability (Figure 2C and Supplemental Figure 8, B–D). To determine the relationship of APOBEC3G with epidermal differentiation and *IFNK* mRNA expression, we analyzed RNA-seq data from monolayer KCs and epidermal raft systems. This showed an inverse relationship with the differentiation stage of both *IFNK* and *APOBEC3G*, with more differentiated KCs having lower expression (Figure 2D). Immunostaining corroborated this, with APOBEC3G expression strongest in the basal layer of the epidermis, colocalizing with IFN-κ (Figure 2E). Consistent with the role of *TMEM173*/STING in regulating IFN responses to CRISPR/Cas9 transfection, we observed significant suppression of *APOBEC3G* mRNA expression in *TMEM173*-KO KCs (Figure 2F and Supplemental Figure 5). These data suggest that STING/IFN-κ–dependent induction of APOBEC3 cytidine deaminases is responsible for CRISPR/Cas9 plasmid degradation in KCs.

### CRISPR/Cas9-generated KO KCs have suppressed IFNK and ISG mRNA expression.

**Figure 3:**
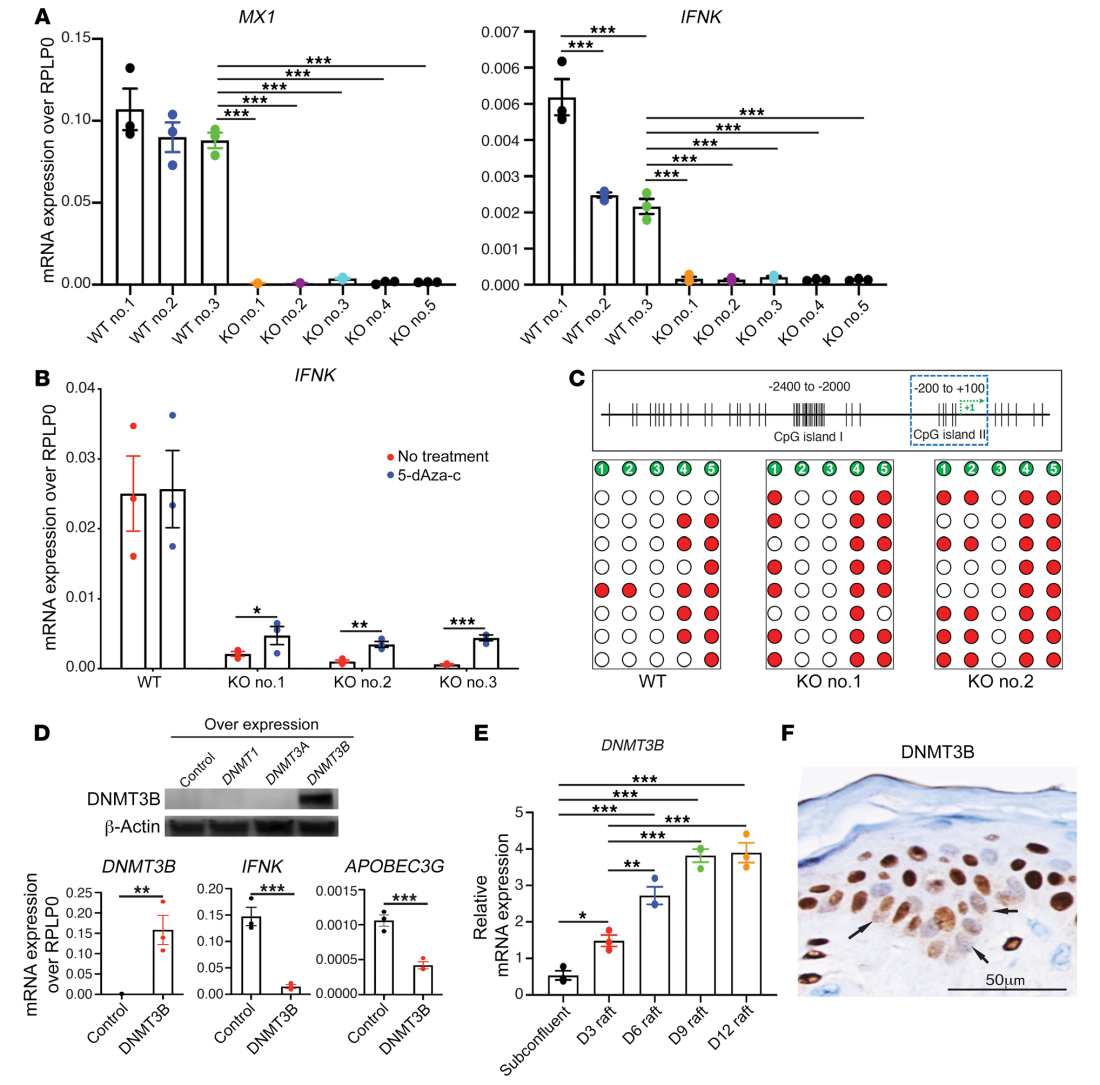
CRISPR/Cas9-generated keratinocyte KOs have suppressed type I IFN responses and *IFNK* expression. (**A**) Decreased *IFNK* expression and type I IFN response (*MX1* expression) in CRISPR/Cas9-generated KO keratinocytes (KCs) (*n* = 3). (**B**) Reversal of *IFNK* expression in CRISPR/Cas9-generated KO KCs after treatment with the demethylating agent 5-dAza-c (*n* = 3). (**C**) CpG hypermethylation in the *IFNK* promoter region in KO KCs (KO 1 and KO 2) compared with nontransfected WT control (*n* = 8). (**D**) Western blot of the DNA methyltransferase DNMT3B in transgenic DNMT1-, DNMT3A-, and DNMT3B-overexpressing KCs (each lane is representative of *n* = 3 independently transfected KCs [upper panel]). Suppression of *IFNK* and *APOBEC3G* mRNA expression in *DNMT3B*-transgenic KCs (lower panel). (**E**) *DNMT3B* mRNA expression in 3D epithelial rafts at different stages of differentiation (*n* = 3). (**F**) DNMT3B protein expression is low in the basal layer (arrows) but increases progressively in the more differentiated layers of the epidermis (*n* = 3). Scale bar: 50 μm. Data in **A**, **B**, **D**, and **E** are represented as mean ± SEM. *P < 0.05; **P < 0.01; ***P < 0.001 by 1-way ANOVA with Tukey’s test (**A** and **E**) or 2-tailed Student’s *t* test (**B** and **D**).

We next examined whether successful transfection of KCs was dependent on repression of *IFNK* expression. Consistent with this notion, we observed that CRISPR/Cas9-generated KOs in KCs have suppressed *IFNK* and ISG mRNA expression, and this was universal across all KC KOs generated, irrespective of the gene target (Figure 3A and Supplemental Figure 9). CpG methylation is a common epigenetic mark for transcriptional regulation (20) and is a known mechanism for controlling *IFNK* expression (21). Therefore, we examined whether CpG methylation could account for *IFNK* and ISG suppression in successfully transfected cells. Treatment of KO KCs with a demethylating agent, 5′-aza-2′-deoxycytidine (5-dAza-C), led to a significant increase in both *IFNK* (Figure 3B) and *MX1* mRNA expression (Supplemental Figure 10) in all KO KCs treated. Additionally, bisulfite sequencing of the *IFNK* promoter revealed a marked increase in CpG methylation in CRISPR KO compared with WT KCs (Figure 3C and Supplemental Figure 11). DNA methyltransferases (DNMTs) are involved in CpG methylation (22) and are expressed in the skin (23). To determine the role of DNMTs in IFN-κ regulation, we generated KCs overexpressing *DNMT1*, *DNMT3A*, or *DNMT3B*. Only *DNMT3B* overexpression led to significant suppression of *IFNK* mRNA expression (Figure 3D), which was accompanied by CpG hypermethylation of the *IFNK* promoter region (Supplemental Figure 12).

We then validated the relevance of this mechanism in vivo. Indeed, *DNMT3B* expression positively correlated with epidermal differentiation, exhibiting the highest expression in fully differentiated epidermal rafts (Figure 3E). Consistent with these findings, *DNMT3B* expression was higher in CRISPR-generated KO compared with WT KCs (Supplemental Figure 13). Confirmatory immunostaining in healthy epidermis showed preferential nuclear expression of the DNMT3B protein in the upper layers of the epidermis, whereas staining was minimal in the lower layers of the epidermis (Figure 3F), where IFN-κ and APOBEC3G were typically colocalized in the basal layer of the epidermis (Figure 2E). These data suggest that DNMT3B is a negative regulator of IFN-κ expression via hypermethylation of the *IFNK* promoter and provide insights into the mechanisms that regulate IFN-κ activity during epidermal differentiation.

### Inhibition of type I IFN autocrine responses through pharmacologic JAK inhibition improves transfection efficiency and prevents generation of low-IFNK-expressing KO KCs.

**Figure 4:**
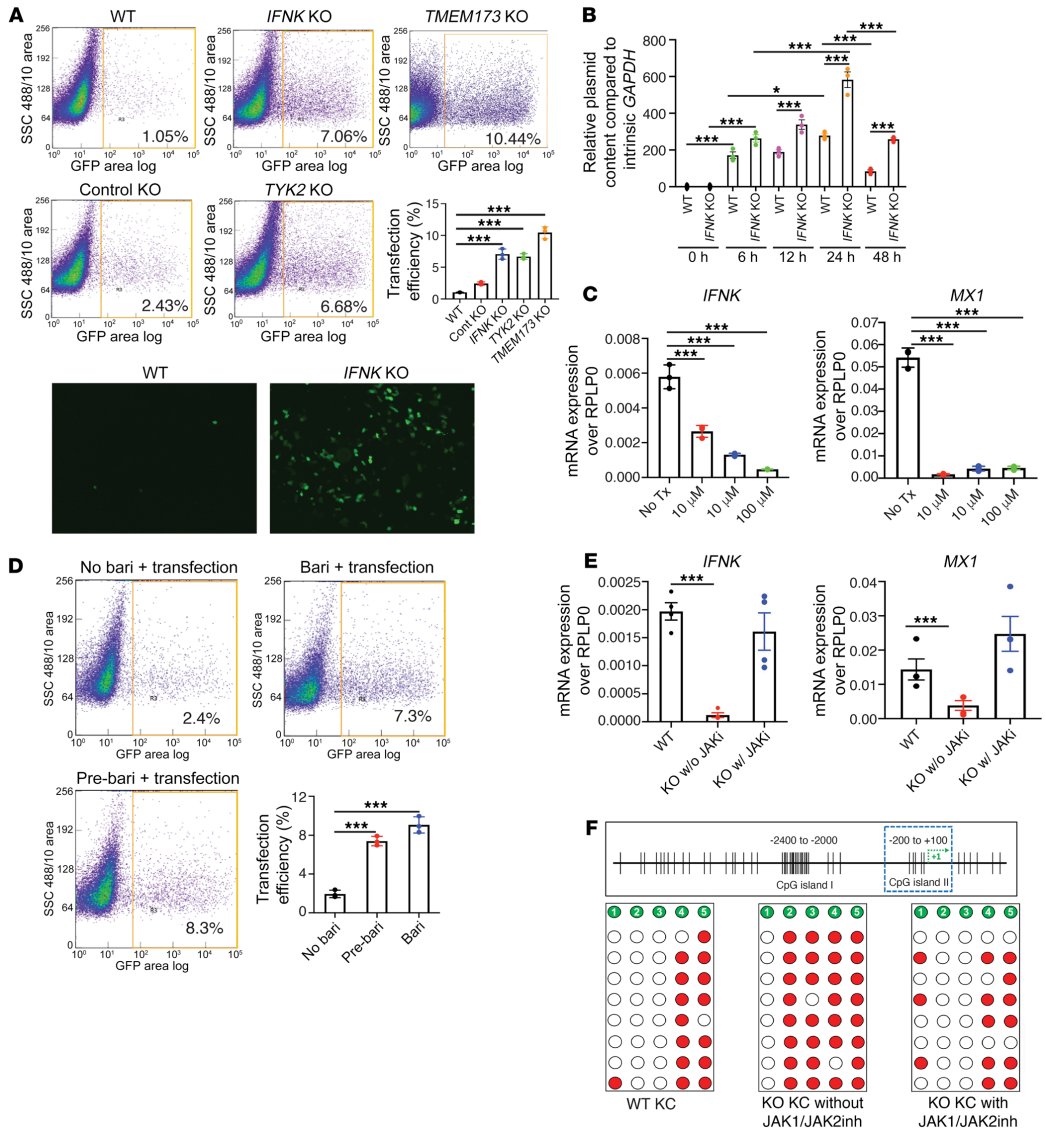
JAK1/JAK2 inhibition prevents suppression of type I IFN response in CRISPR/Cas9-generated KO keratinocytes. (**A**) Increased transfection efficiency in CRISPR/Cas9-generated KO keratinocytes (KCs) (control), and KCs with KO of *IFNK*, *TYK2*, or *TMEM173*, all of which were KOs generated without baricitinib pretreatment (*n* = 3). (**B**) CRISPR/Cas9-generated *IFNK*-KO KCs have increased CRISPR/Cas9 plasmid stability (*n* = 3). (**C**) Suppression of *IFNK* and *MX1* mRNA expression in baricitinib-treated (JAK1/JAK2 inhibitor) KCs (*n* = 3). (**D**) CRISPR/Cas9 transfection efficiency in baricitinib-treated KCs (*n* = 3). (**E**) *IFNK* and *MX1* mRNA expression in the CRISPR/Cas9-generated KO KCs with (w/) or without (w/o) JAK1/JAK2 inhibitor (JAKi). KO KCs with JAK inhibitor (right bar of each of the panels) were selected after JAK inhibitor treatment (*n* = 3). (**F**) CpG methylation in the *IFNK* promoter region in JAK1/JAK2 inhibitor–treated CRISPR KO KCs. KO KCs with JAK inhibitor (right panel) were selected after JAK inhibitor treatment (*n* = 8). Data in **A**–**E** are represented as mean ± SEM. *P < 0.05; **P < 0.01; ***P < 0.001 by 1-way ANOVA with Tukey’s test (**A**–**D**) or 2-tailed Student’s *t* test (**E**).

KC expression of IFN-κ is induced by CRISPR/Cas9 transfection, and IFN-κ directly affects the expression of APOBEC3 cytidine deaminases that in turn promote degradation of intracellular CRISPR/Cas9 plasmids. To determine whether inhibiting type I IFN signaling affects CRISPR/Cas9 transfection efficiency, we used *IFNK*- and *TYK2*-KO KCs to interrupt this IFN autocrine loop. Intriguingly, we observed a marked increase in transfection efficiency (indicated by increased GFP positivity) in both *IFNK*- and *TYK2*-KO KCs. Furthermore, the control KO KCs showed approximately 3-fold increased transfection efficiency compared with WT KCs (Figure 4A), likely due to suppressed IFN-κ autocrine responses (Figure 3A). Consistent with these findings, we also observed increased stability of CRISPR plasmids over time in the *IFNK*-KO KCs (Figure 4B). To validate these findings and determine whether pharmacologic inhibition of Janus kinase (JAK)/IFN signaling would reproduce these findings, we used the JAK1/JAK2 inhibitor baricitinib. Baricitinib effectively decreased mRNA expression of both *IFNK* and the ISG *MX1* in a dose-dependent manner (Figure 4C) and increased transfection efficiency (Figure 4D) to the same level seen in *IFNK*- and *TYK2*-KO KCs (Figure 4A). We reproduced these findings in primary KCs, in which baricitinib significantly increased the transfection efficiency (Supplemental Figure 14). To determine whether IFN-κ affects and promotes selection of low *IFNK*– and ISG–expressing KC KOs, we performed CRISPR/Cas9 transfection in the presence or absence of baricitinib (Supplemental Figure 15). Indeed, *IFNK* and *MX1* expression remained intact in KO KCs generated in the presence of baricitinib (Figure 4E). The reversal of the *IFNK* expression in the KO lines after baricitinib is schematically represented in Supplemental Figure 15. As expected, KO KCs generated in the presence of baricitinib did not have hypermethylation of the *IFNK* promoter region, in stark contrast to KO KCs generated without baricitinib (Figure 4F).

## Discussion

KCs constitute approximately 90% of the cells in the epidermis (24). Given the constant onslaught of external agents and microbiota such as bacteria and viruses, KCs are highly active as sentinels and harbor a range of antimicrobial detectors and pattern recognition receptors for a wide range of pathogens (25). IFN-κ is the predominant type I IFN expressed by KCs and is most prominently expressed in the basal layer of the epidermis (26). The role of this axis in antiviral defenses can be best described in the context of human HPV infections, which are caused by a DNA virus. HPV infections classically involve the mid to upper layers of the epidermis (27), where HPV genome amplification occurs (28). Interestingly, HPVs antagonize the cGAS/STING DNA-sensing pathway to facilitate infection (29). Here, we demonstrate that CRISPR plasmids activate the same type of antiviral response through STING and identify cytidine deaminase APOBEC3G as a key regulator in limiting CRISPR transfection in KCs (Figure 2, B and C).

A surprising observation was that KC KOs generated by CRISPR/Cas9 had permanent suppression of *IFNK* mRNA expression and ISG responses, secondary to *IFNK* promoter hypermethylation. Under normal physiologic conditions in the epidermis, IFN-κ expression is sharply localized to the basal layer of the epidermis, and its expression rapidly decreases in more differentiated layers of the epidermis (26), suggesting that *IFNK* is actively being turned off during the differentiation process. This decrease in *IFNK* coincides with increased expression of the DNA methyltransferase DNMT3B (Figure 3E), which we demonstrate here to be responsible for the *IFNK* promoter hypermethylation in KO KCs, and the subsequent suppression of *IFNK* mRNA expression (Figure 3, C and D, and Supplemental Figure 13). Our data, therefore, suggest that CRISPR transfection is more efficient in cells where IFN-κ has been “turned off” through promoter methylation. This is analogous to HPV infection, which selectively affects KCs that do not express IFN-κ (30), and additionally explains why HPV infections and viral replication predominantly involve mid to upper layers of the epidermis (27). In this context, it is worth noting that DNMT3B expression has been shown to correlate with HPV infection (31, 32), and APOBEC3 members have been shown to restrict HPV infection (33). HPV can also actively suppress IFN-κ expression through the function of the oncogenic proteins E6 and E7 (34, 35), thereby enabling the virus to gain entry into the lower layers of the epidermis where the epidermal stem cells reside.

Prior studies that have looked at immunological processes that may interfere with CRISPR transfection have focused on the potential immunogenicity of the Cas proteins, particularly regarding preexisting adaptive immunity to *Streptococcus pyogenesis* and *Staphylococcus aureus* (36–38). Furthermore, CRISPR gene editing is more efficient in cells that have lost the function of the tumor suppressor p53, as shown in retinal epithelial cells (39), and in human pluripotent stem cells (40). The interplay between CRISPR/Cas9 and intracellular viral sensing pathways has not previously been addressed to our knowledge, and it is likely, based on the data presented here, that this IFN-κ/ABOBEC3G pathway would apply to other transfection systems dependent on DNA-based plasmids, but whether the same mechanism extends to RNA-based transfections remains to be determined.

The use of CRISPR to correct various inherited disorders of the skin holds great promise. The data presented here provide several insights into the molecular mechanisms behind KC transfection resistance, the consequences of which include suppression of IFN responses in genetically corrected KCs, and provides a simple way, with the use of JAK inhibition, to circumvent these issues.

## Methods

### KC culture and treatment.

An immortalized KC cell line, N/TERT (N/TERT-2G) (41), was used with permission from James G. Rheinwald (Brigham and Women’s Hospital, Boston, Massachusetts, USA) for the generation of KO cell lines using nonhomologous end joining via CRISPR/Cas9. This cell line has been shown to have normal differentiation characteristics in both monolayer and organotypic skin models (41). N/TERTs were grown in Keratinocyte-SFM medium (Thermo Fisher Scientific, 17005-042) supplemented with 30 μg/mL bovine pituitary extract, 0.2 ng/mL epidermal growth factor, and 0.3 mM calcium chloride (42). KCs (WT and KO) were treated with a demethylating agent, 5-dAza-C (10 μM; MilliporeSigma, A3656-5MG) for the restoration *IFNK* expression following a previously described protocol (34).

### Generation of CRISPR KO lines in N/TERT KCs.

CRISPR KO KCs were generated as previously described (26). In brief, the sgRNA target sequences were developed (Supplemental Table 1) using a web interface for CRISPR design (https://portals.broadinstitute.org/gpp/public/analysis-tools/sgrna-design). Synthetic sgRNA target sequences were inserted into a cloning backbone, pSpCas9 (BB)-2A-GFP (PX458) (Addgene, 48138), and then cloned into competent *E*. *coli* (Thermo Fisher Scientific, C737303). Proper insertion was validated by Sanger sequencing. The plasmid with proper insertion was then transfected into an immortalized KC line (N/TERTs) using the TransfeX transfection kit (ATCC, ACS4005) in the presence or absence of the JAK1/JAK2 inhibitor baricitinib (10 μg/mL; Advanced Chemblocks, G-5743). GFP-positive single cells were plated and then expanded. Cells were then genotyped and analyzed by Sanger sequencing.

### Generation of overexpressing KC lines.

*DNMT1*-, *DNMT3A*-, and *DNMT3B*-overexpressing KCs were generated by lentiviral transduction of mammalian vectors containing Myc-DDK–tagged human *DNMT1* (Origene, NM_001130823), *DNMT3A* (Origene, NM_175629), and *DNMT3B* (Origene, NM_006892). HEK293T cells were used for viral packaging. Briefly, 10 μg of expression vector was mixed with an equal amount of packaging plasmid (Origene, TR30037) in 1 mL Opti-MEM medium (Invitrogen, 31985062) and 30 μL Turbofectin (Origene, TF8100), and incubated at room temperature for 5 minutes. The obtained mixture was added to the HEK293T cells without dislodging the cells. Supernatants from infected HEK293T cells were harvested after 24 hours, filtered using 0.45 μm syringe filters, aliquoted, and stored at –80°C or used immediately. N/TERT KCs were plated 1 day before transduction in serum- and antibiotic-free medium. The next day, the cells were transduced at a multiplicity of infection of 0.5 along with 8 μg/mL polybrene (Sigma-Aldrich, TR-1003-G). Twenty-four hours after transfection, media were replaced with complete growth media. Cells were passaged the following day and the medium containing puromycin was used from then on. Puromycin concentration for KCs was determined by performing a drug-kill curve. An empty mammalian expression vector containing the Myc-DDK tag only (Origene, PS100001) was used as a negative control for transduction experiments. Nontransduced cells were also treated with puromycin to observe complete cell death. Once all the cells in control wells were killed, limited dilution was performed to obtain single cells that were expanded, and the clones were verified for overexpression by Western blotting.

### RNA extraction and qRT-PCR.

RNA extraction, qRT-PCR, and RNA-seq were performed following a protocol we published previously (26). RNAs were isolated from cell cultures using a Qiagen RNeasy plus kit (catalog 74136). qRT-PCR was performed on a 7900HT Fast Real-time PCR system (Applied Biosystems) with TaqMan Universal PCR Master Mix (Thermo Fisher Scientific) and TaqMan probes (*IFNK*, Hs00737883_m1; *MX1*, Hs00895608_m1; *TMEM173*, Hs00736955_g1; *APOBEC3A*, Hs02572821_s1; *APOBEC3B*, Hs00358981_m1; *APOBEC3C*, Hs00819353_m1; *APOBEC3D*, Hs00537163_m1; APOBEC3F, Hs01665324_m1; *APOBEC3G*, Hs00222415_m1; *APOBEC3H*, Hs00419665_m1; *FLG*, Hs00856927_g1: *CCL5*, Hs00982282_m1; *CXCL10*, Hs00171042_m1; *IFIT2*, Hs01584837_s1; *IFNL1*, Hs00601677_g1; *IRF7*, Hs01014809_g1; *DNMT3B*, Hs00171876_m1; *IFNB1*, Hs01077958_s1; *TREX1*, Hs03989617_s1; *DNASEI*, hs00173736_m1; *DNASEII*, Hs00172391_m1).

### scRNA-seq from human skin.

Generation of single-cell suspensions for scRNA-seq was performed as follows from the normal human epidermis. Samples were incubated overnight in 0.4% Dispase (Life Technologies) in Hank’s balanced saline solution (Gibco) at 4°C. Epidermis and dermis were separated. The epidermis was digested in 0.25% Trypsin-EDTA (Gibco) with 10 U/mL DNase I (Thermo Fisher Scientific) for 1 hour at 37°C, quenched with FBS (Atlanta Biologicals), and strained through a 70 μm mesh. The dermis was minced, digested in 0.2% collagenase II (Life Technologies) and 0.2% collagenase V (Sigma-Aldrich) in plain medium for 1.5 hours at 37°C, and strained through a 70 μm mesh. Epidermal and dermal cells were recombined, and libraries were constructed by the University of Michigan Advanced Genomics Core on the 10× Genomics Chromium system. Libraries were then sequenced on the Illumina NovaSeq 6000 sequencer to generate 151-bp paired-end reads. Data processing including quality control, read alignment, and gene quantification were conducted using the 10× Genomics Cell Ranger software. Seurat was used for normalization, data integration, and clustering analysis (43). Clustered cells were mapped to corresponding cell types by matching cell cluster gene signatures with putative cell-type-specific markers. The scRNA-seq data can be found in the NCBI Gene Expression Omnibus (GEO GSE179162).

### scATAC-seq from human skin.

Skin biopsies (4 mm) were obtained from the palm/hip of a healthy individual. Biopsies were then incubated in 0.4% Dispase overnight to separate the epidermis and dermis. After the separation, the epidermis was transferred to 0.25% Trypsin-EDTA with 10 U/mL DNase mixture and incubated at 37°C for 1 hour. The epidermis mixture was then quenched with FBS and precipitated by centrifugation. Cell pellets were then resuspended in PBS with 0.04% BSA. Cell numbers were counted at this step for future dilution calculation. The nuclei isolation protocol was carried out as described by 10× Genomics. Of note, cells obtained from the epidermis were incubated in lysis buffer on ice for 7 minutes to achieve the optimal lysis efficiency. The cell lysis efficiency was determined by Countess II FL Automated Cell Counter. The scATAC-seq library was prepared by the Advanced Genomics Core at the University of Michigan. Ten thousand nuclei per sample and 25,000 reads per nuclei were targeted, and the libraries were sequenced using a NovaSeq SP 100 cycle flow cell. The raw data were first processed by the Chromium Single Cell ATAC Software Suite (10× Genomics), and then analyzed using the Signac package in R. Briefly, the scATAC-seq data underwent a serial of analyses, including quality control, dimension reduction, clustering, and integration with previously annotated scRNA-seq data. The DNA accessibility profile was then visualized in different cell types and samples. The scATAC-seq data can be found in the NCBI Gene Expression Omnibus (GEO GSE226926).

### Accell siRNA knockdown.

N/TERT KCs were plated in 96-well plates (30,000 cells/well) and incubated at 37°C with 5% CO_2_ overnight. Accell siRNA (100 μM; Dharmacon: *APOBEC3A*, E-017432-00-0005; *APOBEC3B*, E-017322-01-0005; *APOBEC3G*, E-013072-00-0005; *APOBEC3H*, E-019144-00-0005; *DNASE1*, E-016280-00-0005) was prepared in 1× siRNA buffer (Dharmacon, B-002000-UB-100). One microliter of 100 μM siRNA was diluted with 100 μL Accell delivery medium (Dharmacon, B-005000) for each well of 96-well plates. The growth medium was removed from the cells and 100 μL of the appropriate delivery mix with siRNA was added to each well and the plate was incubated at 37°C with 5% CO_2_. Accell Non-targeting Control siRNA (Dharmacon, D-001910-01-05) was used as a negative control. After 72 hours, cells were harvested for RNA preparation. RNA isolation and qRT-PCR were as described above.

### 3D human epidermal tissue cultures.

Normal human epidermal KCs were isolated from a pool of neonatal foreskins (*n* = 3) and grown using J2-3T3 mouse fibroblasts as feeder layer as originally described by Rheinwald and Green (44). 3D human epidermal raft cultures seeded in collagen hydrogels were prepared using 3 distinct donor pools as described previously (45) and grown at an air-liquid interface for 12 days in E-Medium (DMEM/DMEM-F12 [1:1], 5% FBS, 180 μM adenine, 5 μg/mL bovine pancreatic insulin, 5 μg/mL human apo-transferrin, 5 μg/mL triiodothyronine, 4 mM L-glutamine, 10 ng/mL cholera toxin, 10 μg/mL gentamicin, and 0.25 μg/mL amphotericin B). After 9 days at an air-liquid-interface to allow for epidermal maturation, the reconstructed human epidermises were treated with 0.1% BSA/PBS (Sigma-Aldrich) as vehicle control or 10 ng/mL TNF-α, IL-17A, and IL-22 (R&D Systems) alone or in combination for 72 hours, harvested, and analyzed for changes in gene expression as described previously (46). Epidermal tissues were separated from the collagen scaffold and lysed in QIAzol (Qiagen) for RNA isolation. RNA-seq and analyses were performed according to the methods described above.

### Measurement of CRISPR plasmid stability in KCs.

CRISPR plasmid (PX458, Addgene, 48138) was transfected into KCs using a Transfex transfection kit (ATCC, ACS-4005). Cells were then harvested at different time points (0, 6, 12, 24, and 48 hours) and washed with PBS 3 times to remove extracellular plasmid from the cells. The DNA was then purified using the QIAamp DNA Blood Mini kit (Qiagen, 51106). CRISPR plasmid–specific primers (GFP-F1, GGAGAGGGCAGAGGAAGTCT and GFP-R1, GAACTTCAGGGTCAGCTTGC) were used to perform qPCR with the DNAs isolated from the transfected KCs using SYBR Green PCR Master Mix (Thermo Fisher Scientific, 4309155) on the 7300 Real-Time PCR System (Applied Biosystems).

### Transfection efficiency measurement.

Different cell types such as KCs, fibroblasts, and HEK293T cells were grown in their respective culture medium and the cells were then transfected with either GFP-linked CRISPR plasmid (PX458 from Addgene) or non-CRISPR plasmid (pCMV-GFP from Addgene) using a Transfex kit (ATCC) or FuGene6 (Promega). Cells were then kept in a CO_2_ incubator for 24 hours. Cells were then harvested using 0.25% Trypsin-EDTA and then resuspended in PBS. GFP-positive cells were then analyzed at the University of Michigan Flow Cytometry core. We consider the percentage of GFP-positive cells as the transfection efficiency of the respective cell types.

### Bisulfite sequencing analysis of the IFNK promoter.

Based on the vendor’s recommendations, bisulfite treatment was performed on DNA isolated from WT and CRISPR KO KCs using the EZ DNA Methylation-Gold kit (Zymo Research, D5005). Bisulfite-converted DNA was amplified with the following primers: IFNK-BS-F9 (TGTTGGGATGGATTATTTAGGTATT) and IFNK-BS-R9 (TTCAACAAAAAAAATTTTCTCATTC). PCR products were cloned in pCR2.1-TOPO vector (Thermo Fisher Scientific, K204040) and those clones were then subjected to Sanger sequencing using M13Rev and T7 primers.

### Western blotting.

Total protein was isolated from cells using Pierce RIPA buffer (Thermo Fisher Scientific, 89900) with PMSF protease inhibitor (Sigma-Aldrich, 36978) and run in precast gels (Bio-Rad, 456-1094S). The membrane was blocked with 3% BSA and then probed with primary antibodies against the proteins p-IRF3 (Thermo Fisher Scientific, PA536775), IRF3 (Abcam, ab68481), p-STING (Cell Signaling Technology, 19781S), p-STAT1 (Thermo Fisher Scientific, 33-3400), DNMT3B (Cell Signaling Technology, 67259S), and β-actin (Sigma-Aldrich, A5441), followed by secondary antibodies (anti-mouse or -rabbit IgG, AP-linked, Cell Signaling Technology), washed 3 times, and substrate added (Thermo Fisher Scientific, 45-000-947). They were then developed with a chemiluminescence kit and imaged on an iBright imager (Thermo Fisher Scientific).

### Immunostaining.

Formalin-fixed, paraffin-embedded tissue slides obtained from healthy individuals were heated for 30 minutes at 60°C, rehydrated, and epitope retrieved with Tris-EDTA (pH 6). Slides were blocked and incubated with primary antibodies against IFN-κ (Abnova, H00056832-M01), APOBEC3G (Abcam, Ab223704), and DNMT3B (Cell Signaling Technology, 67259S) overnight at 4°C. Slides were incubated with biotinylated secondary antibodies (biotinylated goat anti-rabbit IgG antibody, Vector Laboratories, BA1000; biotinylated horse anti-mouse IgG antibody, Vector Laboratories, BA2000) and then incubated with fluorochrome-conjugated streptavidin. Slides were prepared in mounting medium with 4′,6-diamidino-2-phenylindole (DAPI) (VECTASHIELD Antifade Mounting Medium with DAPI, H-1200, VECTOR). Images were acquired using an inverted Zeiss microscope. Images presented are representative of at least 3 biologic replicates.

### Statistics.

Data were analyzed using GraphPad Prism 9.0. Data are represented as the mean ± SEM. The numerical results between 2 groups were analyzed by a 2-tailed Student’s *t* test and multiple comparisons were analyzed by 1-way ANOVA with post hoc Tukey’s test.

### Study approval.

Human skin samples were obtained under University of Michigan IRB–approved protocol (HUM00151834), and all participants consented to a 6-mm punch biopsy. The study was conducted according to the Declaration of Helsinki principles.

## Author contributions

MKS and JEG conceptualized the study. MKS, RU, AK, XX, S Shao, OP, S Sirobhushanam, EX, and YJ performed experiments and analyzed data. MKS developed the CRISPR/Cas9 method in keratinocytes. CZ, ACB, and LCT analyzed scATAC-seq and scRNA-seq data. BEPW provided RNAs from 3D raft keratinocytes. MKS, JEG, LCT, and JMK acquired funding for this research. MKS and JEG wrote the original draft of the manuscript. MKS, RU, CZ, ACB, LCT, AK, XX, BEPW, S Shao, OP, S Sirobhushanam, EX, YJ, KAG, JJV, JMK, and JEG reviewed and edited the manuscript.

## Supplementary Material

Supplemental data

## Version 1 03/16/2023

In-Press Preview

## Version 2 05/01/2023

Electronic publication
