# Supplementary material for: Keratinocytes sense and eliminate CRISPR DNA through STING/IFN-κ activation and APOBEC3G induction
Source: J Clin Invest. 2023 May 1;133(9):e159393. doi: 10.1172/JCI159393 (PMC10145927; doi:10.1172/JCI159393)
Supplement: Supplemental data [file jci-133-159393-s136.pdf]

Supplemental Table 1

List of the genes and their sgRNAs used to generate KO keratinocytes

IFNK (KO#1): GTTCAGTAAGTTACAGTCCA
TYK2 (KO #2): GACTCACTGAAAGTGACCCA
BATF2 (KO # 3): CTTCTGCCGGCTTCGCTGGG
PLA2G2F (KO # 4): GTAGCAACCGTAGCCCACGA
IL17RA (KO # 5): TCCCCGTGGCTCACATCGAA

qPCR primers (for SYBR Green):

GFP-F1: GGAGAGGGCAGAGGAAGTCT
GFP-R1: GAACTTCAGGGTCAGCTTGC
GAPDH-F: GGAAGGTGAAGGTCGGAGTC
GAPDH-R: GAAGATGGTGATGGGATTTC

Supplemental Figure 1

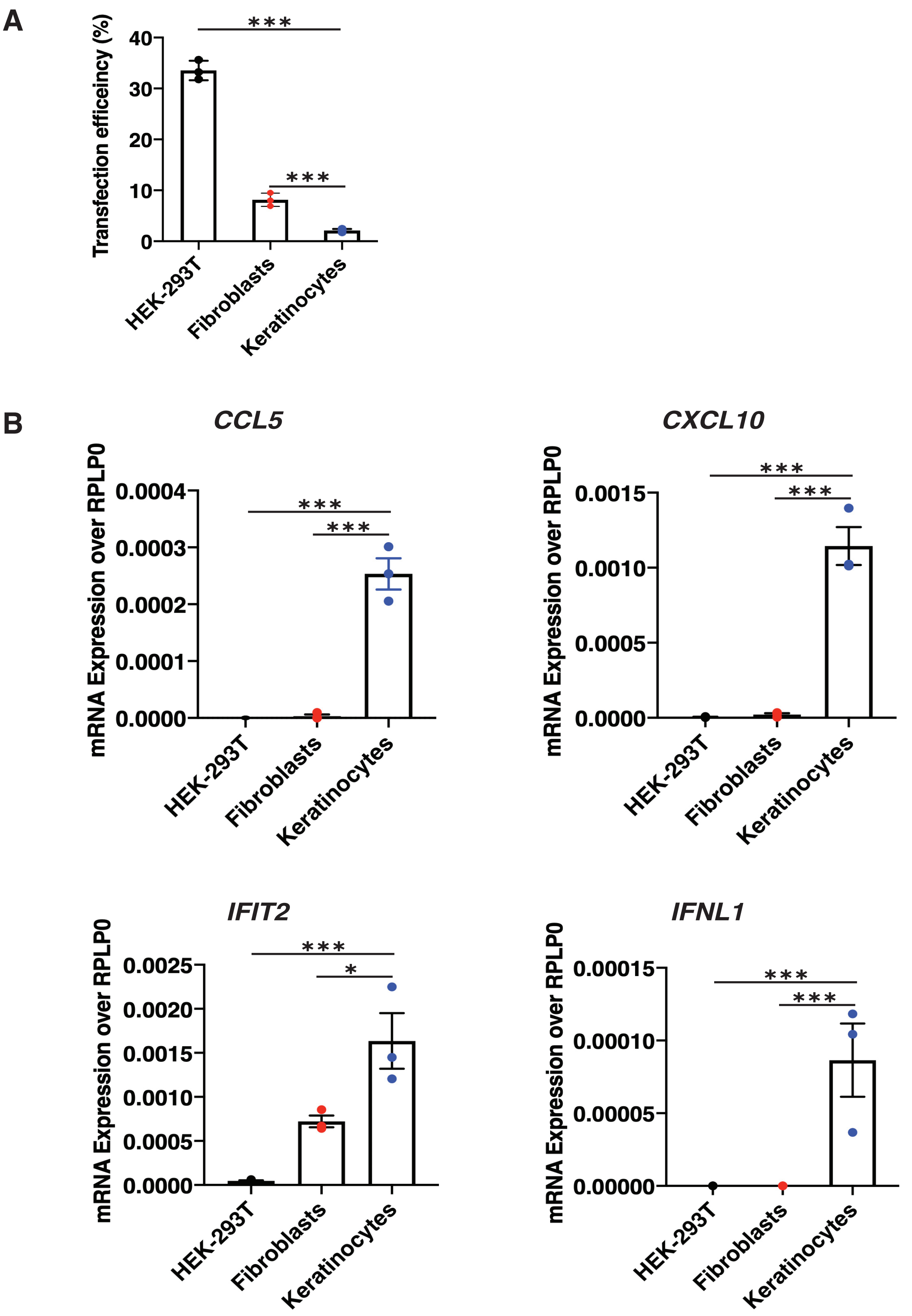

Supplemental Figure 1. (A) Transfection efficiency in keratinocytes along with fibroblasts and HEK293T cells using non-CRISPR plasmid (pCMV-GFP) and FuGENE6 transfection reagent. (B) *CCL5*, *CXCL10*, *IFIT2* and *IFNL1* expression in keratinocytes, fibroblasts and HEK293T cells. (Data are represented as mean ± SEM; *P < 0.05,***P < 0.001, 1-way ANOVA with Tukey's test; n=3).

Supplemental Figure 2

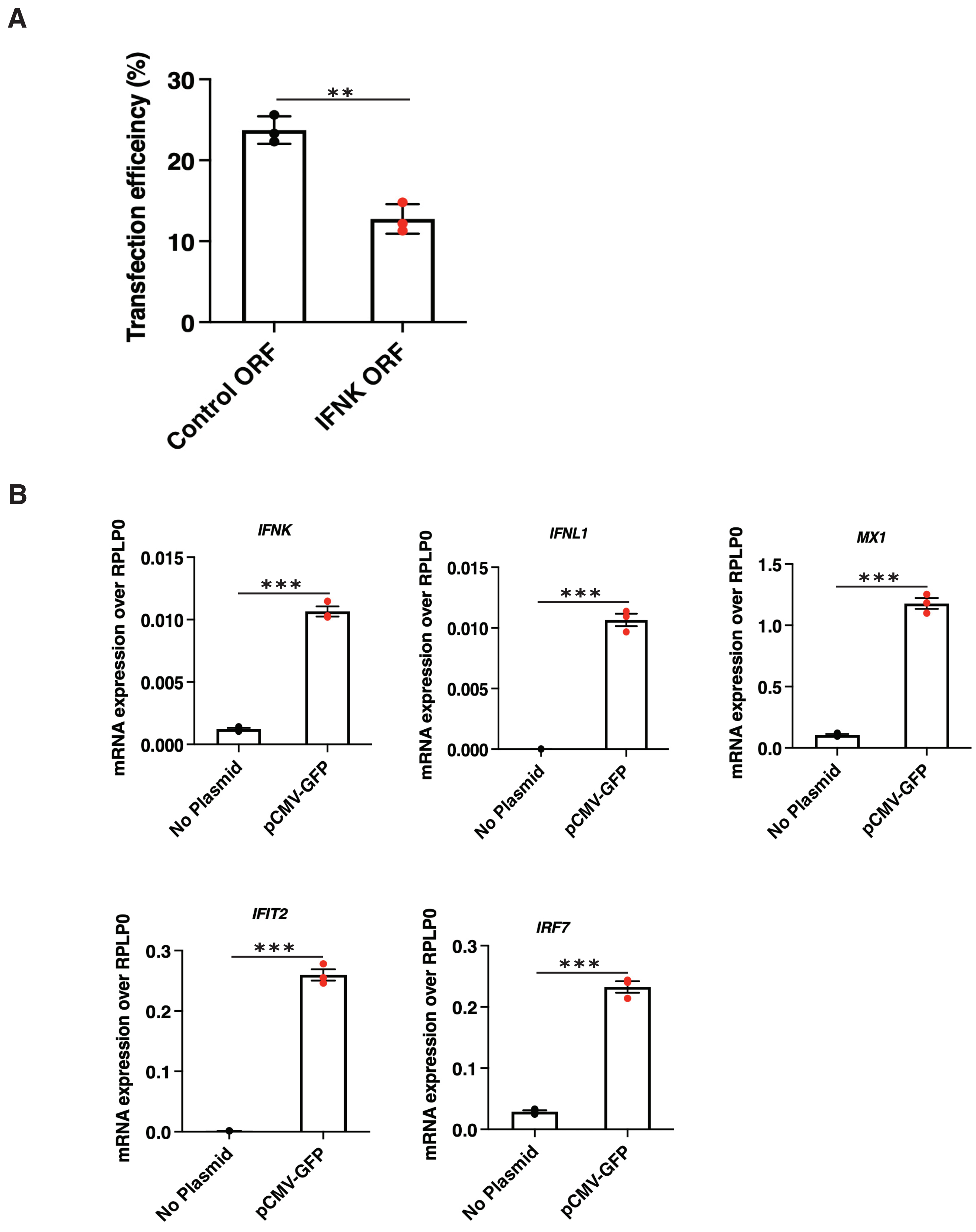

Supplemental Figure 2. (A) Transfection efficiency in IFNK overexpressed (*IFNK* ORF, GenScript # OHu13357) HEK293T Cells. (B) IFNK and other ISGs expression in non-CRISPR plasmid (pCMV-GFP) treated keratinocytes. (Data are represented as mean ± SEM; **$P < 0.01$, ***$P < 0.001$, 2-tailed Student's t-test; n=3).

Supplemental Figure 3

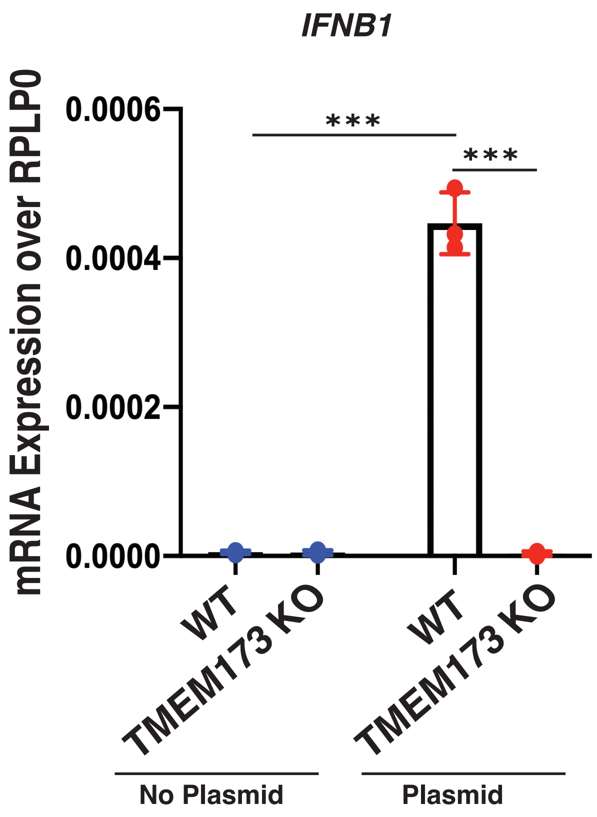

Supplemental Figure 3. *IFNB1* expression in CRISPR plasmid treated keratinocytes. *IFNB1* expression in CRISPR plasmid treated WT and *TMEM173* (STING) KO keratinocytes (Data are represented as mean ± SEM; ***$P < 0.001$, 1-way ANOVA with Tukey's test; n=3). Bars with blue dots: no treatment; bars with red dots: CRISPR plasmid treatment.

Supplemental Figure 4

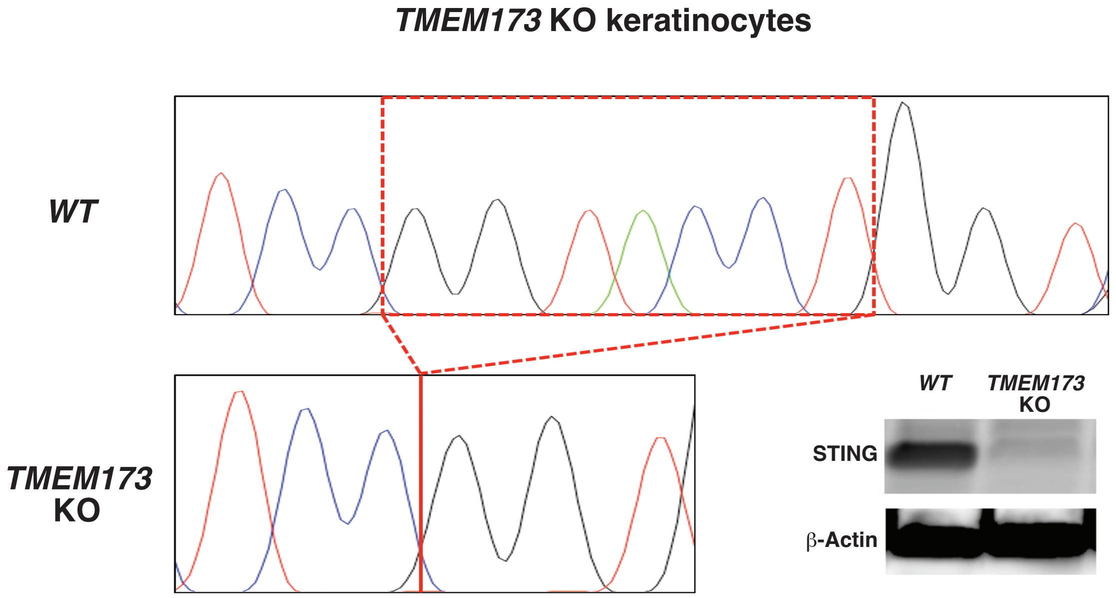

Supplemental Figure 4. Chromatogram and western blot for *TMEM173* KO keratinocytes. TMEM173 (STING protein) KO keratinocytes were generated by CRISPR-Cas9. Chromatogram shows homozygous mutation with 7 nucleotides deletion. STING western blot in TMEM173 KO KCs.

Supplemental Figure 5

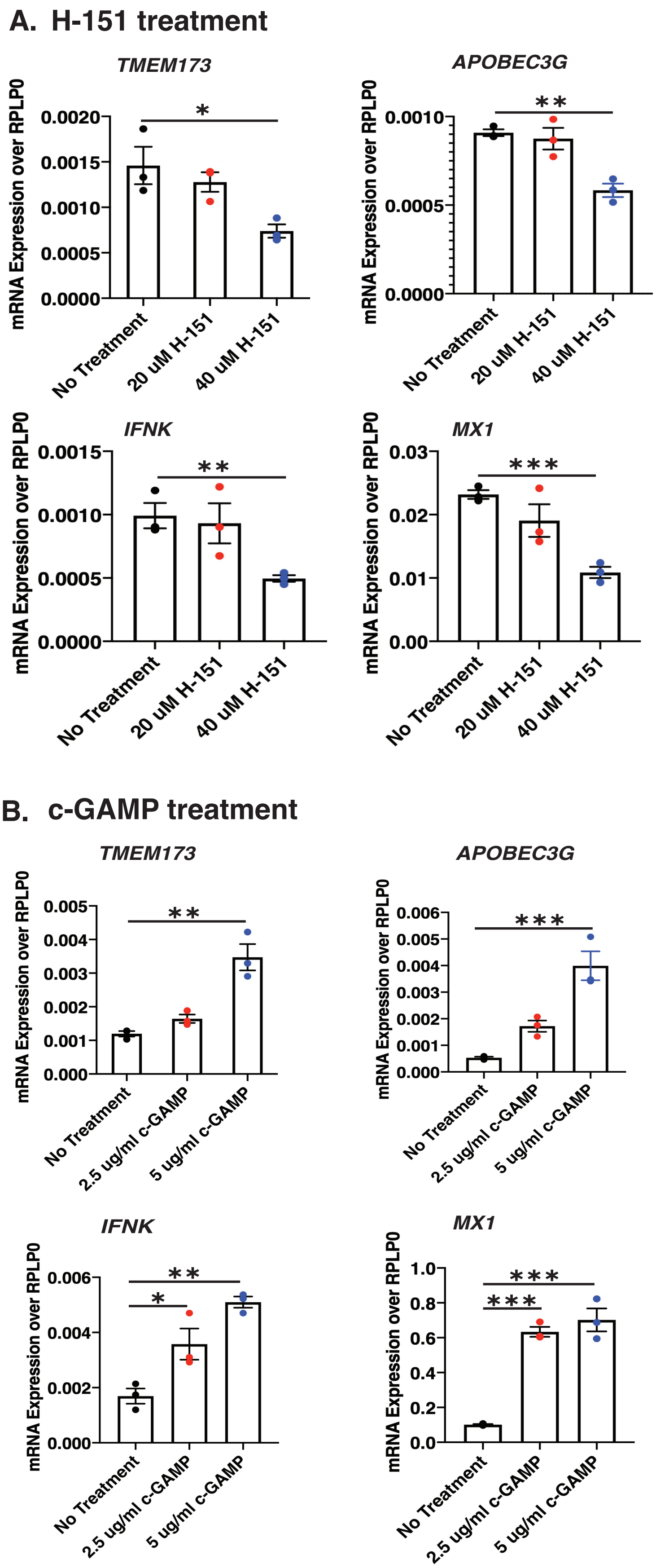

Supplemental Figure 5. *APOBEC3G* mRNA expression and type I IFN response in presense of STING inhibitor (H-151) (A) and STING activator (c-GAMP) (B). (For **A**, data are represented as mean ± SEM; *P < 0.05, **P < 0.01, ***P < 0.001, 2-tailed Student's t-test; n=3, and for **B**, data are represented as mean ± SEM; *P < 0.05, **P < 0.01, ***P < 0.001, 1-way ANOVA with Tukey's test; n=3).

Supplemental Figure 6

A

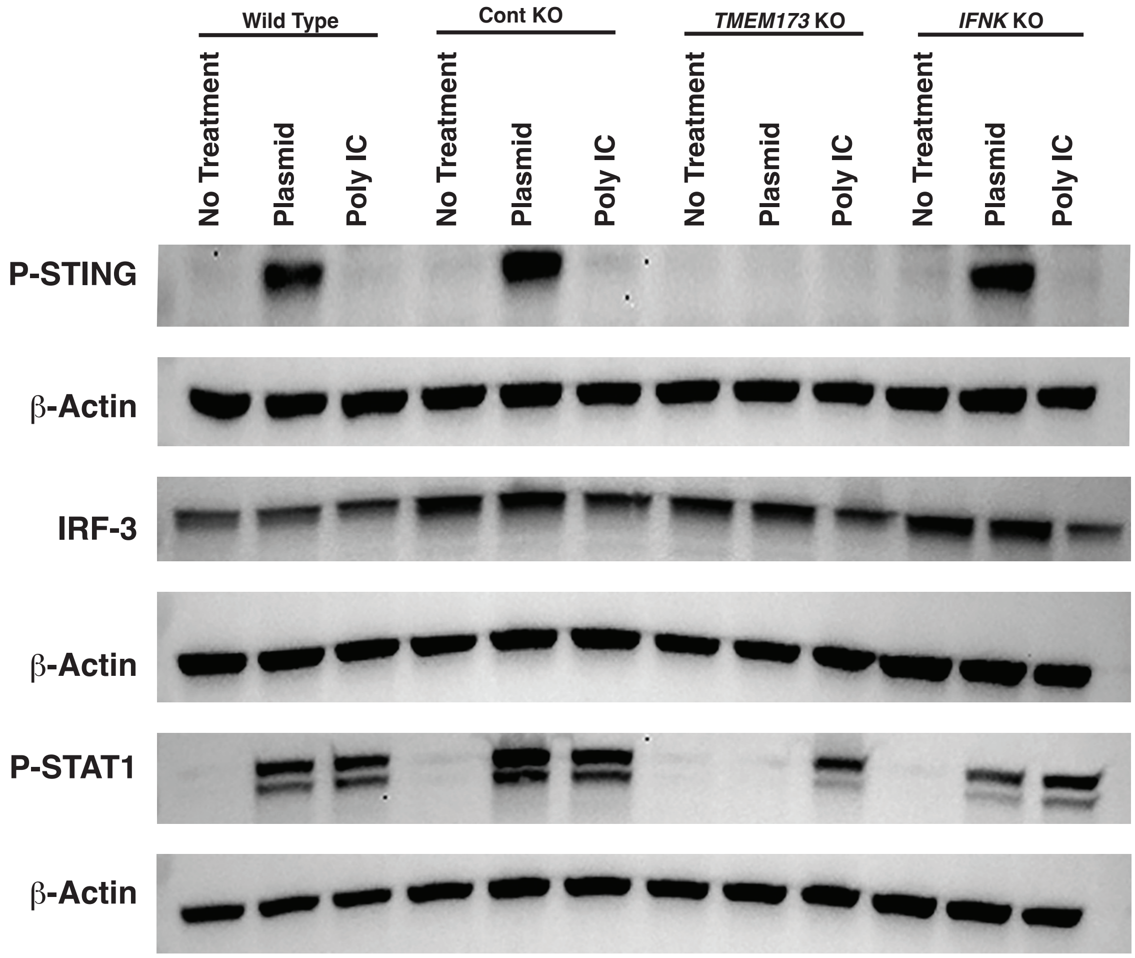

B

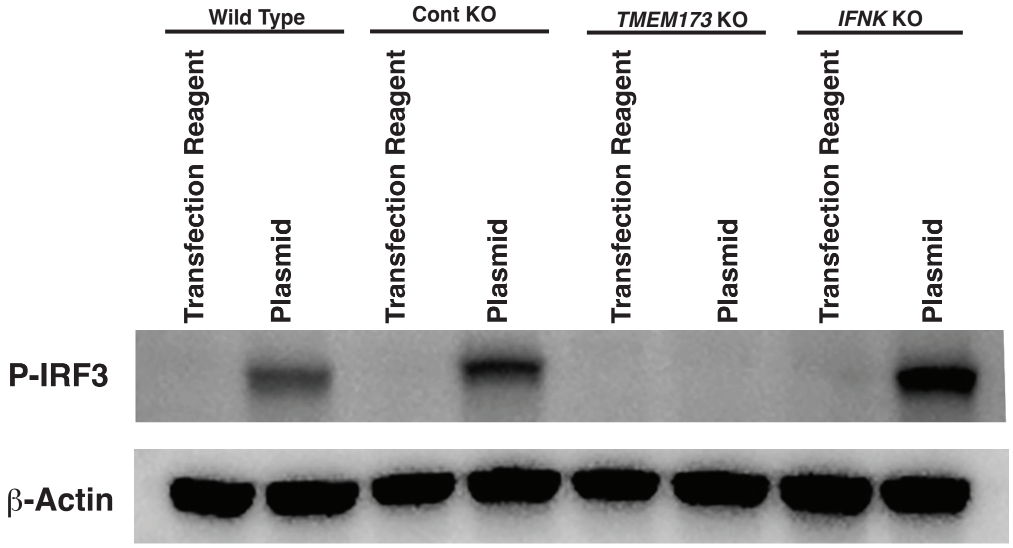

Supplemental Figure 6. (A) P-STING (S366), IRF-3 and P-STAT1 (Y701) western blots were performed in WT, Control CRISPR KO, TMEM173 KO and IFNK KO keratinocytes with the treatment of CRISPR plasmid (PX458). Poly IC was used as positive control. (B) P-IRF3 wesstern blot was performed with transfection reagent only. CRISPR plasmid was used here as postive control.

Supplemental Figure 7

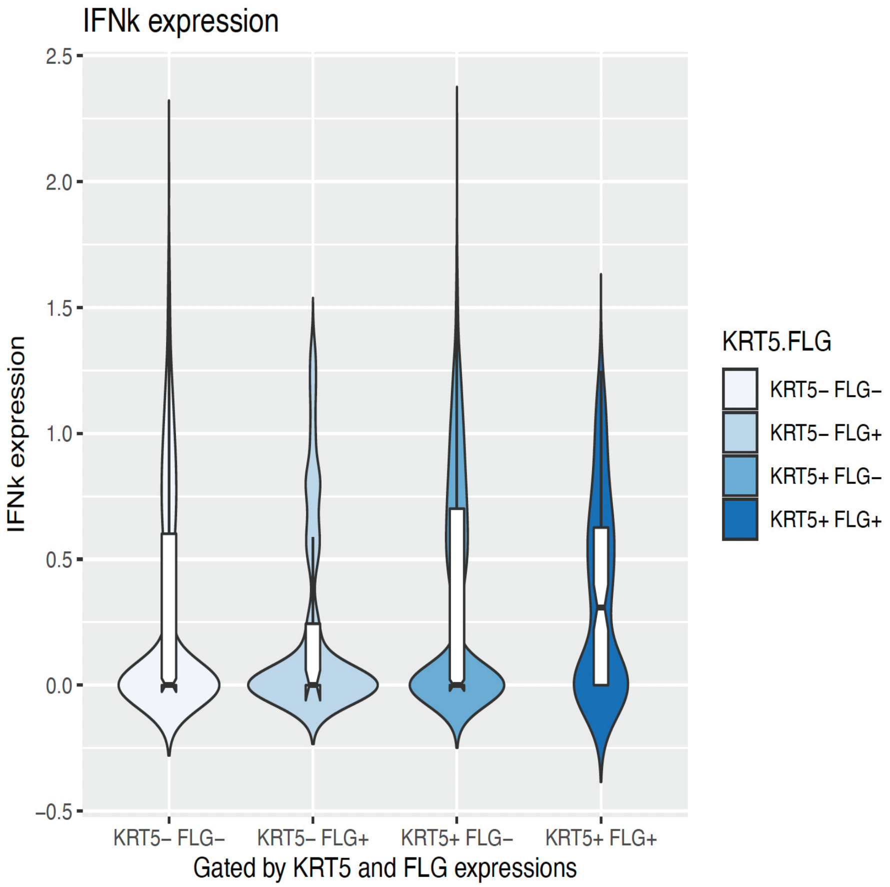

Supplemental Figure 7: *IFNK* expression in single-cell ATAC-seq from healthy human epidermis gated by KRT5 and FLG expression. This Violin plot showing comparison of *IFNK* expression in all the different conditions (KRT5-FLG-; KRT5-FLG+; KRT5+FLG- and KRT5+FLG+).

Supplemental Figure 8

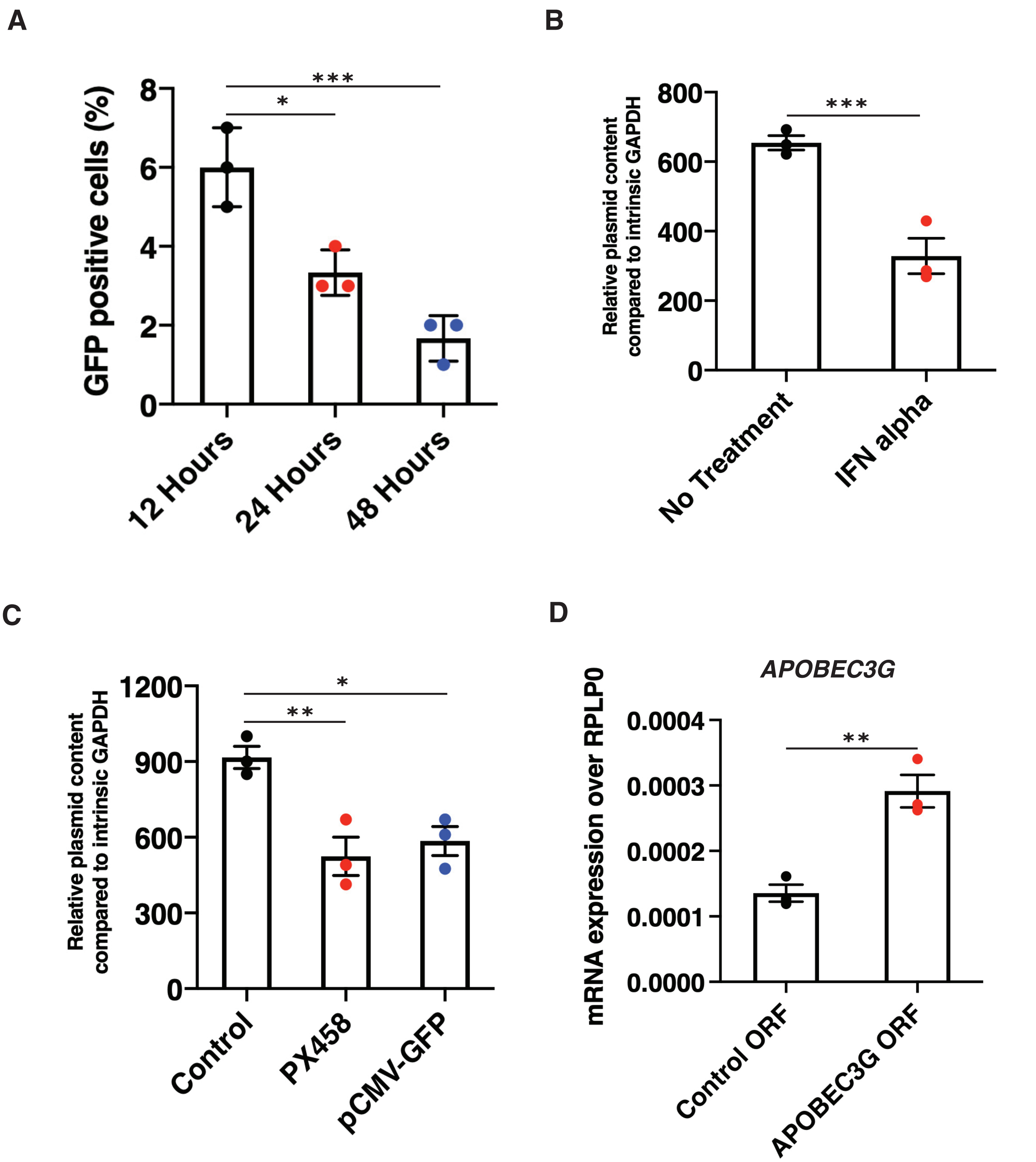

Supplemental Figure 8. (A) Percentage of GFP postive cells at different time point after non-CRISPR plasmid transfection (pCMV-GFP). (B) Plasmid stability in keratinocytes after IFN alpha treatment (10 ng/ml). (C) Plasmid stability (CRISPR Plasmid PX458 and a non-CRISPR plasmid pCMV-GFP) in APOBEC3G overexpressed HEK293T cells. (D) APOBEC3G overexpression was valiadted by quantitaive PCR in overexpressed HEK293T cells.(For **A** and **C**, data are represented as mean ± SEM; *P < 0.05, **P < 0.01, ***P < 0.001, 1-way ANOVA with Tukey's test; n=3 and for **B** and **D**, data are represented as mean ± SEM; **P < 0.01, ***P < 0.001, 2-tailed Student's t-test; n=3).

Supplemental Figure 9

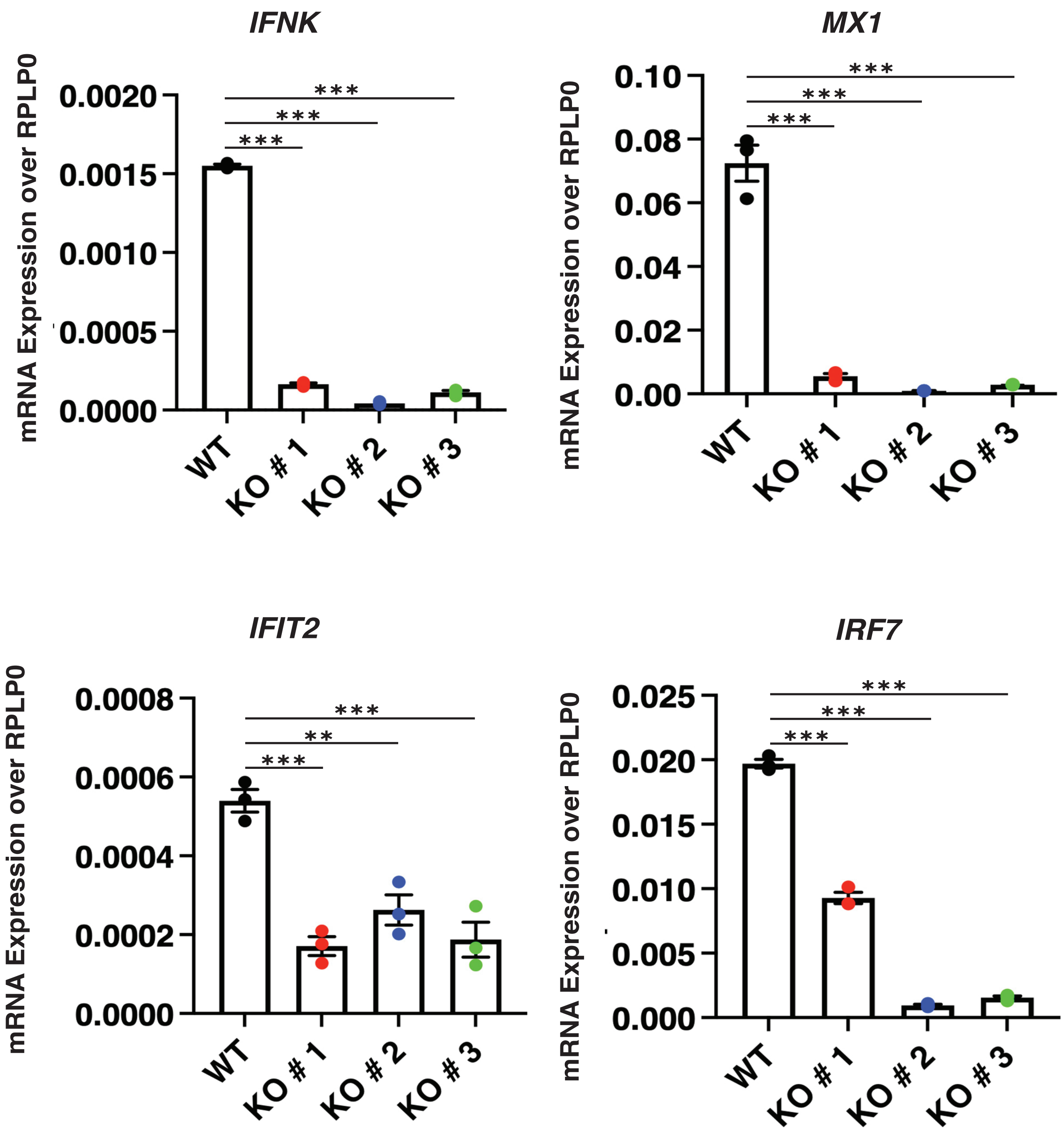

Supplemental Figure 9. CRISPR-cas9 generated keratinocytes KOs using FuGENE6 have suppressed type I IFN responses and IFNK expression. (Data are represented as mean ± SEM; $**P < 0.01$, $***P < 0.001$, 1-way ANOVA with Tukey's test; n=3). KO # 1, KO # 2 and KO # 3 are three independent KO kertinocytes.

Supplemental Figure 10

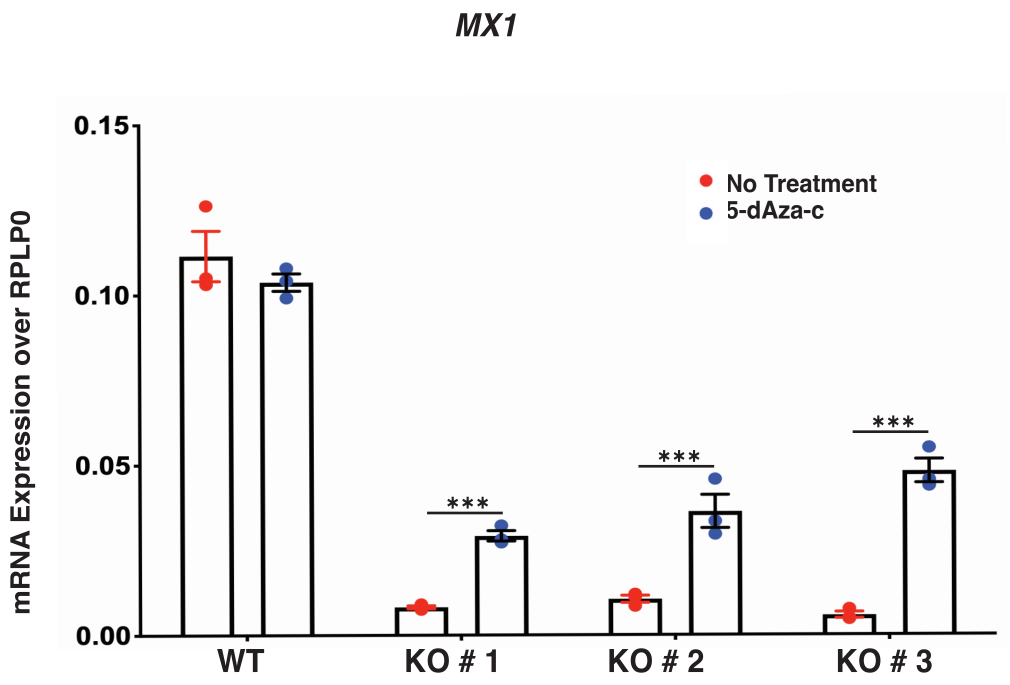

Supplemental Figure 10. MX1 expression in CRISPR-cas9 generated KO keratinocytes after treatment with the demethylating agent 5-dAza-c. (Data are represented as mean ± SEM; ***P < 0.001, 2-tailed Student’s t-test; n=3).

Supplemental Figure 11

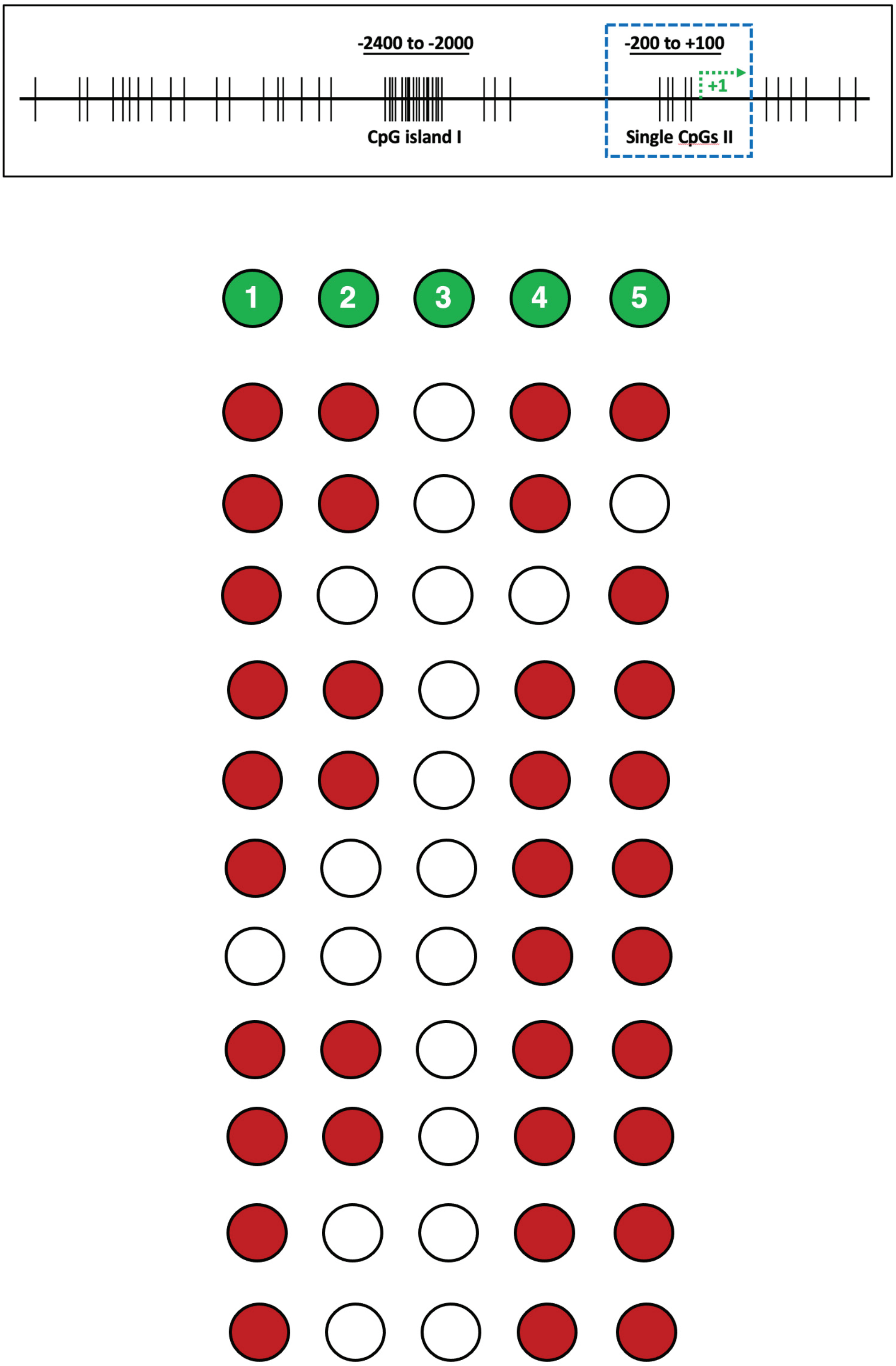

Supplemental Figure 11. CpG methylation analysis in I*FNK* promoter region in a polyclonal population of CRISPR/Cas9 plasmid selected all GFP positive cells (n=11)**.**

Supplemental Figure 12

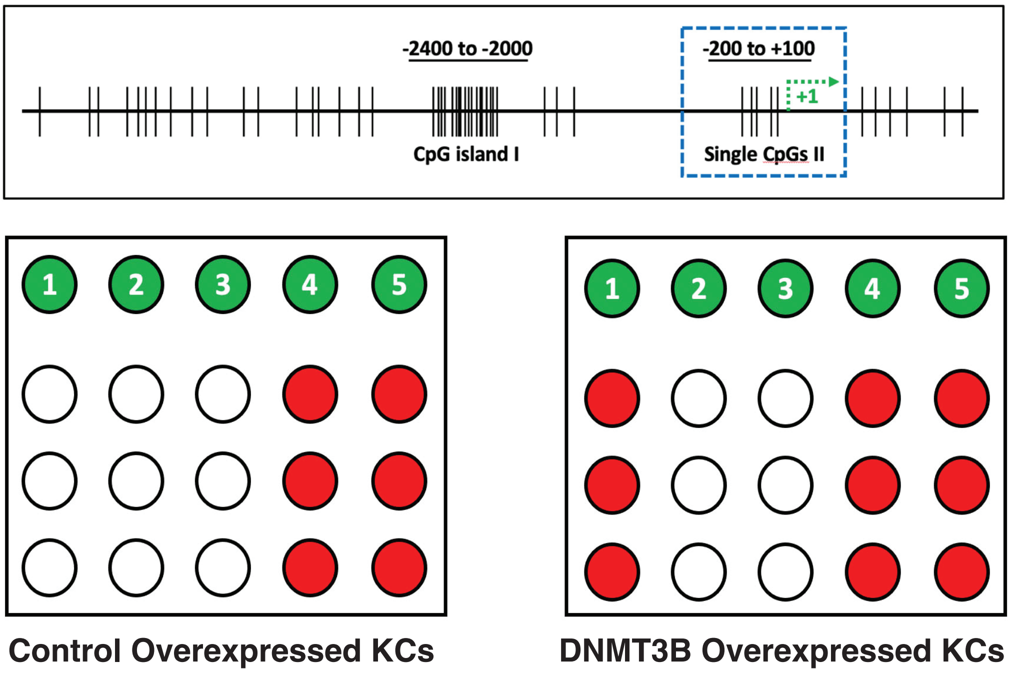

Supplemental Figure 12. CpG methylation analysis in DNMT3B overexpressed keratinocytes. CpG hypermethylation in the *IFNK* promoter region in the DNMT3B overexpressed compared to control overexpressed keratinocytes (n=3).

Supplemental Figure 13

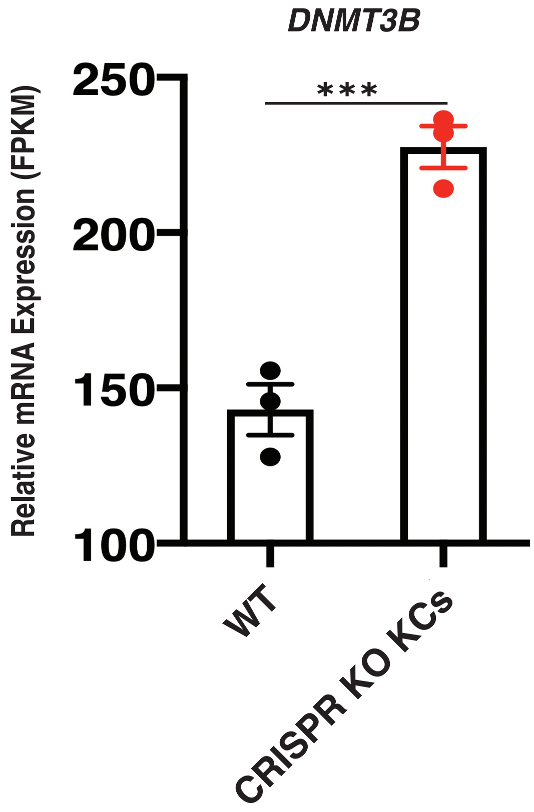

Supplemental Figure 13. DNMT3B expression in CRISPR KO keratinocytes. (Data are represented as mean ± SEM; ***P < 0.001, 2-tailed Student's t-test; n=3).

Supplemental Figure 14

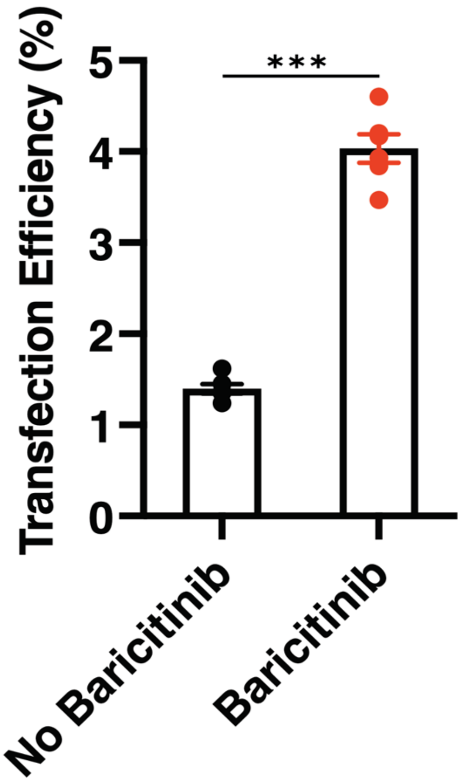

Supplemental Figure 14. CRISPR Transfection efficiency in primary keratinocytes. (Data are represented as mean ± SEM; ***$P < 0.001$, 2-tailed Student's t-test; n=6).

Supplemental Figure 15

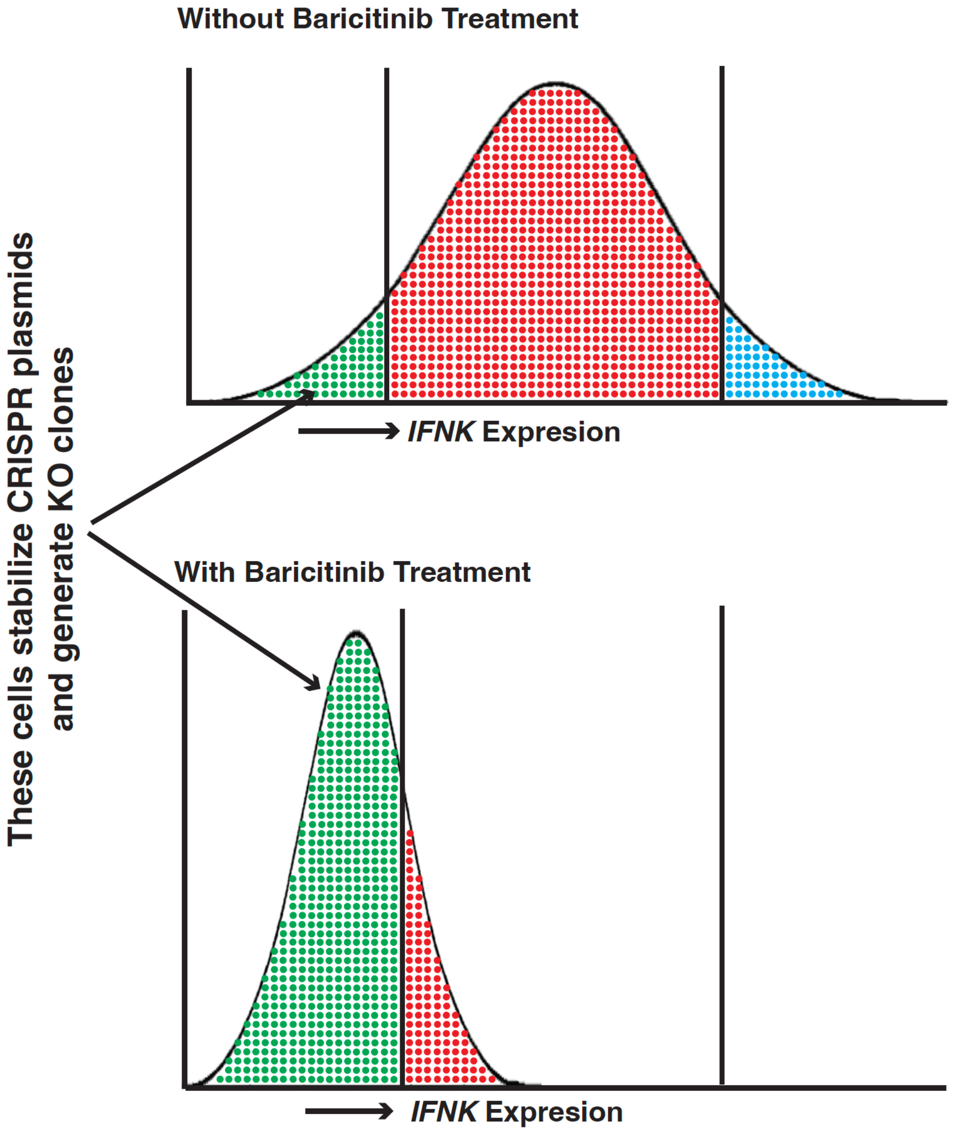

Supplemental Figure 15. Schematics to show keratinocytes with suppressed *IFNK* selected for CRISPR transfection. CRISPR plasmids select only low *IFNK* expressing keratinocytes (green dots in the upper panel) and eventually generate KO lines with suppressed *IFNK* whereas baricitinib treatment suppresses *IFNK* expression in the significant population (green dots in the lower panel) and those cells are selected for CRISPR transfection and generate KO lines with regained *IFNK* expressions (Figure 4E).
